# Dissociation between gut microbiota remodeling and early gut-liver injury in CLP-induced sepsis

**DOI:** 10.3389/fphar.2026.1800088

**Published:** 2026-04-20

**Authors:** Tijana Mašić, Aleksandra Ružičić, Siniša Đurašević, Tanja Jevdjović, Iva Lakić, Slavica Borković-Mitić, Slađan Pavlović, Jovan Jevtić, Katarina Kruščić, Ivica Dimkić, Nebojša Jasnić

**Affiliations:** 1 Department of Animal and Human Physiology, Institute for Physiology and Biochemistry “Ivan Đaja”, Faculty of Biology, University of Belgrade, Belgrade, Serbia; 2 Department of Physiology, Institute for Biological Research “Siniša Stanković” - National Institute of the Republic of Serbia, University of Belgrade, Belgrade, Serbia; 3 Institute of Pathology, Faculty of Medicine, University of Belgrade, Belgrade, Serbia; 4 Department of Biochemistry and Molecular Biology, Institute for Physiology and Biochemistry “Ivan Đaja”, Faculty of Biology, University of Belgrade, Belgrade, Serbia

**Keywords:** cecal ligation and perforation (CLP), gut microbiota, gut-liver axis, metabolic preconditioning, organ injury, sepsis

## Abstract

**Introduction:**

Sepsis is a life-threatening condition in which early host responses critically determine organ dysfunction, yet strategies targeting this critical window remain limited. We investigated whether pre-septic modulation of host metabolism and gut microbiota could mitigate early organ injury in severe polymicrobial sepsis.

**Methods:**

Male Sprague-Dawley rats were pretreated for 4 weeks and then subjected to cecal ligation and perforation (CLP). Outcomes were assessed within the first 24 h after sepsis induction, with survival monitored for 7 days. Gut microbiota composition was analyzed at the group level before and after CLP.

**Results:**

Early sepsis was characterized by adrenal catecholamine depletion, ileal villus shortening, colonic inflammatory activation, rapid gut microbiota restructuring, and hepatic oxidative stress with selective inflammatory transcriptional activation. Although pretreatments altered baseline gut microbiota composition and partially preserved commensal and short-chain fatty acid-associated taxa, they did not improve survival nor prevent early intestinal and hepatic injury. Early mortality occurred exclusively in meldonium-pretreated animals, indicating a potential trade-off of metabolic preconditioning under severe septic stress. Despite pretreatment-specific modulation of selected antioxidant enzymes, hepatic redox imbalance and stress-associated protein oxidation persisted during early sepsis.

**Discussion:**

Collectively, these observations indicate an apparent dissociation at the group level between gut microbiota remodeling and early gut-liver injury. The first 24 h after sepsis onset thus emerge as a period of limited pharmacological plasticity, underscoring the need for therapeutic strategies that directly target the robust, systemic, host-driven stress mechanisms predominating during this early phase in severe sepsis.

## Graphical Abstract

**Figure:**
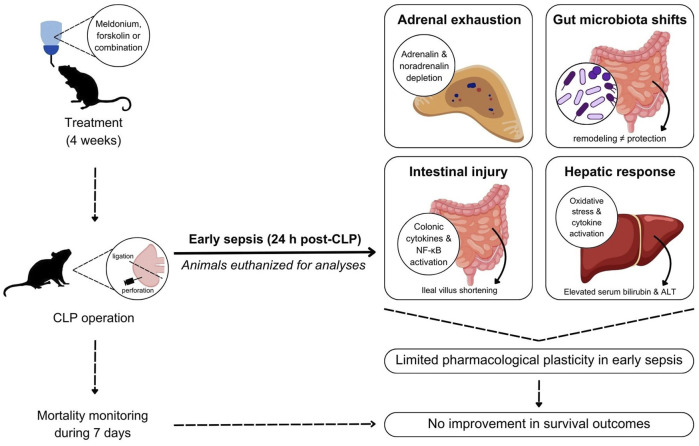

## 1 Introduction

Sepsis is a life-threatening condition caused by a dysregulated host response to infection which, if not rapidly controlled, leads to organ dysfunction and high mortality (Singer et al., 2016; van der Poll et al., 2021). Early sepsis progression is marked by rapid systemic changes, with the first 24 h representing a critical period during which organ injury trajectories are established and outcomes are largely determined (Raghavan and Marik, 2006; Zhang et al., 2024). Despite advances in supportive care, effective strategies targeting early pathophysiological mechanisms remain limited (Srdić et al., 2024). Although pharmacological approaches targeting inflammation, metabolism, and the microbiota have been proposed as potentially beneficial in sepsis, increasing evidence suggests that these mechanisms may be pharmacologically refractory during the early phase of the disease (van der Poll et al., 2021; Srdić et al., 2024).

Among the organs affected during early sepsis, the intestine and liver play central and interconnected roles. Disruption of intestinal barrier integrity facilitates the translocation of microbial and inflammatory signals (Niu and Chen, 2021; Sun et al., 2022), while the liver acts as a primary sensor and integrator of these inputs, regulating metabolic adaptation and innate immune responses (Sun et al., 2020). Perturbation of this gut-liver axis has been implicated in the amplification of systemic inflammation and early organ dysfunction during sepsis (Zhang et al., 2022).

Accumulating experimental and clinical evidence indicates that the host response to sepsis depends strongly on the physiological state at the time of insult (Prescott et al., 2019; Gerard et al., 2025). Sepsis can lead to divergent inflammatory and survival outcomes depending on the baseline host conditions, as shown by both experimental pretreatment models and clinical cohort studies (Ashare et al., 2005; Alrawashdeh et al., 2022). These observations suggest that early sepsis progression is determined primarily by the host state rather than pathogen burden alone (Prescott et al., 2019). This concept provides a framework for exploring how pre-septic modulation of host metabolism influences early sepsis development (Tan et al., 2019; Iske et al., 2024). Importantly, early sepsis is increasingly recognized as a phase of limited pharmacological plasticity, in which host-driven neuroendocrine and oxidative stress responses may override classical inflammatory modulation (Prescott et al., 2019; Husabø et al., 2020).

Sepsis involves early and profound disturbances in cellular energy metabolism that contribute to organ dysfunction (Wasyluk and Zwolak, 2021; Liu et al., 2022). Meldonium and forskolin are pharmacological agents with distinct effects on host energy regulation (Dambrova et al., 2016; Salehi et al., 2019). Meldonium inhibits carnitine-dependent mitochondrial fatty acid oxidation and alters lipid metabolic balance, thereby limiting metabolic flexibility under increased energetic demand (Đurašević et al., 2019; Đurašević et al., 2021b). Notably, in our previous experimental sepsis models, meldonium pretreatment was associated with increased early mortality despite reported anti-inflammatory, antioxidative ant anti-apoptotic properties (Đurašević et al., 2021a; Đurašević et al., 2022; Ružičić et al., 2025), suggesting that metabolic restriction may compromise adaptive host responses during severe systemic stress. In contrast, forskolin promotes lipolysis and β-oxidation while modulating inflammatory signaling through activation of adenylate cyclase and increasing intracellular cAMP (El-Agroudy et al., 2016; Zhang et al., 2019), with additional reported effects on gut microbiota composition (Tung et al., 2021).

Based on this framework, we hypothesized that pre-septic modulation of host energy metabolism would influence the trajectory of early host responses to sepsis. We therefore selected meldonium and forskolin as metabolic modulators with distinct mechanisms of action in order to examine how metabolic preconditioning shapes responses to polymicrobial sepsis induced by cecal ligation and puncture (CLP). Focusing on the first 24 h after sepsis induction, we aimed to assess the effects of such modulation on survival, gut microbiota composition, intestinal and hepatic responses, inflammatory signaling, and oxidative stress, as well as to identify whether specific early pathophysiological processes remain resistant to such modulation, particularly within the gut-liver axis.

## 2 Materials and methods

### 2.1 Animals and ethical approval

All animals were obtained from the animal facility of the Faculty of Biology, University of Belgrade, Republic of Serbia. Eight-week-old male Sprague-Dawley rats weighing 400.6 ± 43.3 g were housed two per cage for 4 weeks with *ad libitum* access to a standard diet (Veterinary Institute, Subotica, Serbia) and tap water. The animals were acclimated to 22 °C ± 1 °C and maintained under a 12 h light/dark regime. Animals were randomly assigned to experimental groups using a simple randomization procedure. Due to the nature of the experimental interventions, blinding of group allocation during treatment was not feasible. However, outcome assessments, including histological and molecular analyses, were performed without knowledge of group assignment whenever possible.

All procedures involving animals were performed in accordance with the Serbian Animal Welfare Law, Directive 2010/63/EU, and the ARRIVE guidelines and 3R principles. Following the National legislation, all animal procedures were approved by the Veterinary Directorate of the Ministry of Agriculture, Forestry, and Water Management (permit No. 003508964 2024).

### 2.2 Experimental treatments and induction of CLP sepsis

Analytical-grade meldonium was obtained from Kono Chem Co., Ltd. (China), while *Coleus forskohlii* extract containing 10% forskolin was purchased from Xi’an Lyphar Biotech Co., Ltd. (China). Rats had *ad libitum* access to tap water or tap water supplemented with meldonium, forskolin, or their combination. Water intake was measured three times per week, and treatment compound concentrations were adjusted accordingly to ensure the intended dosage. Based on previously reported doses capable of modulating metabolic pathways without over toxicity meldonium (300 mg/kg/day) and/or forskolin (100 mg/kg/day) were administered during the 4-week treatment period (Ríos-Silva et al., 2014; Majeed et al., 2015; Đurašević et al., 2019; Đurašević et al., 2021b).

The experimental groups were organized as follows: a sham control group (SHAM) that underwent a sham operation procedure (n = 16), a sepsis group (S) that underwent the CLP operation procedure (n = 20), an S + M group (SM) that received meldonium before the CLP operation (n = 20), an S + F group (SF) that received forskolin before the CLP operation (n = 20), and an S + M + F group (SMF) that received both meldonium and forskolin before the CLP operation (n = 22) (Table 1). After 4 weeks of treatment, all groups of animals were subjected to surgery.

**TABLE 1:** Experimental group design and treatments.

Groups	Meldonium in water	Forskolin in water	Sham surgery	CLP surgery	n
SHAM	-	-	+	-	16
S	-	-	-	+	20
SM	+	-	-	+	20
SF	-	+	-	+	20
SMF	+	+	-	+	22

Rats were divided into five experimental groups based on pre-treatment (tap water, meldonium, forskolin, or both) and type of surgical procedure (sham or cecal ligation and puncture, CLP). n = number of animals per group.

**FIGURE 1:**
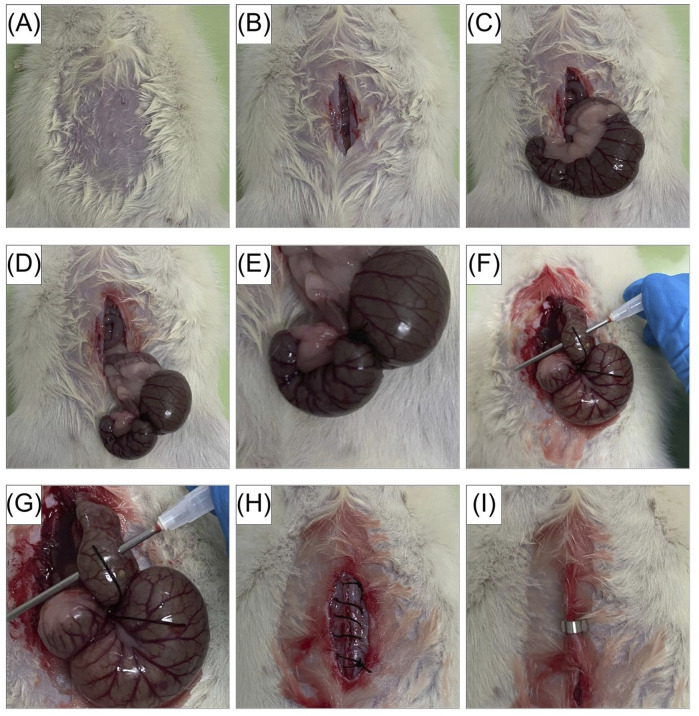
Cecal ligation and puncture (CLP) procedure in rats. **(A)** Abdominal area shaved and disinfected. **(B)** A midline incision (∼2 cm) was made. **(C)** The cecum was exteriorized. **(D,E)** Approximately 40% of the cecum was ligated below the ileocecal valve using 3-0 silk. **(F,G)** The ligated cecum was punctured three times with a 16-gauge needle, and a small amount of fecal content was gently extruded. **(H,I)** The cecum was returned to the peritoneal cavity, and the abdominal wall and skin were closed.

Sepsis was induced by CLP, a well-established model that recapitulates key features of human polymicrobial sepsis (Buras et al., 2005). A severe CLP protocol was applied, as previously described (Rittirsch et al., 2009), to capture the early, high-risk phase of disease. Briefly, rats were anesthetized with ketamine (0.08 mL/100 g body weight) and xylazine (0.05 mL/100 g body weight). A 2-cm midline laparotomy was performed to expose the cecum. Approximately 40% of the cecum was ligated below the ileocecal valve using a 3-0 silk suture and punctured three times with a 16-gauge needle. A small amount of fecal material was gently extruded to ensure patency, after which the cecum was returned to the abdominal cavity and the incision was closed (Figure 1). Rats in the SHAM group underwent the identical laparotomy and cecal exposure without ligation or puncture.

### 2.3 Tissue collection and survival analysis

At 24 h after surgery, a subset of animals from each experimental group was euthanized by rapid decapitation using a stainless-steel guillotine in accordance to recommendations of FELASA (Federation of European Laboratory Animal Science Associations). Blood, liver, ileum, and colon were collected; intestinal segments were gently flushed with saline to remove luminal contents. Liver tissue and distal colon were immediately stored at −80 °C for subsequent analyses, while ileum and proximal colon were processed for histopathological examination, and blood samples were used for serum isolation. The remaining animals were monitored for an additional 7 days to assess survival. Survival analysis was performed using the Kaplan-Meier method.

### 2.4 Fecal microbiota analysis

#### 2.4.1 Fecal sample collection

Fecal samples were collected at two time points: after the 4-week treatment period but immediately before the operation (pre-operation, 0 h), and 24 h after the operation (post-operation, 24 h). Given the coprophagic behavior of rats, one composite fecal sample per cage was collected at each time point. The collected samples were pooled at the group level, homogenized, and divided into three aliquots, which were processed and analyzed as parallel technical replicates. Based on this sampling approach, the experimental groups for microbiota analysis were defined as follows: pre-operation control group (SHAM/S_0h), pre-operation meldonium-septic group (SM_0h), pre-operation forskolin-septic group (SF_0h), pre-operation meldonium-forskolin-septic group (SMF_0h), post-operation sham control group (SHAM_24h), post-operation septic group (S_24h), post-operation meldonium-septic group (SM_24h), post-operation forskolin-septic group (SF_24h), and post-operation meldonium-forskolin-septic group (SMF_24h). Accordingly, microbiota analyses reflect cage-level, group-based ecological shifts rather than individual-level biological variability, and should be interpreted within this experimental framework.

#### 2.4.2 DNA extraction and amplicon sequencing preparation

Fecal DNA was extracted using the ZR Fecal/Soil DNA Miniprep Kit (Zymo Research, United States) following the manufacturer’s instructions. The V3–V4 hypervariable regions of the bacterial 16S rRNA gene were amplified using the primers CCTAYGGGRBGCASCAG (forward) and GGACTACNNGGGTATCTAAT (reverse). PCR amplification, library preparation, and sequencing were performed by Novogene Co., Ltd., using proprietary protocols optimized for Illumina NovaSeq PE250 sequencing.

#### 2.4.3 Bioinformatics processing and statistical analysis

Raw paired-end reads were processed locally using QIIME2 (v2024.10) in a Linux environment (Ubuntu/WSL) (Bolyen et al., 2019). Quality filtering, trimming, denoising, merging, and chimera removal were performed with DADA2 (Callahan et al., 2016), producing amplicon sequence variants (ASVs). Taxonomy was assigned using a naïve Bayes classifier trained on the SILVA reference database (v138.1), trimmed to the V3–V4 region (Quast et al., 2012). Feature table, taxonomy, and metadata were exported from QIIME2 and imported into R (v4.5.2) using phyloseq and qiime2R. ASVs lacking phylum-level annotation were removed, and only taxa present in ≥5% of samples were retained. Rarefaction was applied to an even depth of 50,903 reads per sample (rarefy_even_depth). Alpha diversity (Observed ASVs, Shannon index) was calculated from the rarefied table in phyloseq. Group differences were assessed by one-way ANOVA and Tukey’s *post hoc* test (p < 0.05). Beta diversity was calculated using Bray–Curtis dissimilarities. Principal coordinates analysis (PCoA) was performed, and 2D ordination plots were generated. Overall differences between groups were tested using PERMANOVA (adonis2, 999 permutations), followed by pairwise comparisons with Benjamini–Hochberg correction. For taxonomic composition, counts were aggregated to the Genus level, converted to relative abundance, and taxa representing <2% on average within a group were grouped as “represented <2%”. Relative-abundance bar plots were generated using ggplot2, and ANCOM-BC2 was applied to identify differentially abundant taxa (p < 0.05) among the experimental groups.

### 2.5 Histopathological analysis of the ileum and colon

After animal sacrifice, ileum and colon tissue samples were collected and immediately fixed in 10% neutral buffered formalin for 24 h. Following fixation, the samples were rinsed with distilled water and subsequently dehydrated through a graded series of ethanol solutions (from 70% to absolute alcohol). After dehydration, the samples were cleared in xylene and embedded in paraffin blocks. Paraffin sections were cut at a thickness of 3–5 μm using a standard microtome. The obtained sections were stained with hematoxylin and eosin (H&E) for histological evaluation.

The stained slides were scanned using the Leica Aperio AT2 digital slide scanner. Morphometric measurements were performed using the Aperio ImageScope software (Leica Biosystems, United States). For each animal, the following morphometric parameters were analyzed: villus height, villus width, crypt height, crypt width, mucosal thickness, submucosal thickness, muscular layer thickness, and degree of inflammation. The degree of inflammation was evaluated based on the presence of inflammatory cells in different parts of the tissue, which was scored as 0 (absent), 1 (serosa), 2 (mucosa), 3 (submucosa), and 4 (lamina propria). For each parameter, ten independent measurements were obtained, and the mean value was calculated and used for subsequent analysis. Data were statistically analyzed according to the procedure detailed in Section 2.6.6.

### 2.6 Molecular and biochemical analyses

#### 2.6.1 Measurement of serum liver function parameters

Blood was collected and incubated at room temperature for 45 min, and then centrifuged at 2000× g for 10 min at 4 °C. The resulting supernatant was transferred into clean tubes and stored at −80 °C until further analysis.

To evaluate liver function, serum levels of total proteins, albumin, and bilirubin were measured, along with the activities of alkaline phosphatase (ALP), alanine aminotransferase (ALT), aspartate aminotransferase (AST), lactate dehydrogenase (LDH), and gamma-glutamyl transferase (GGT). All parameters were quantified using BioSystems reagents (Barcelona, Spain) on a Mindray BS-240 Vet Auto Chemistry Analyzer (Shenzhen Mindray Animal Medical Technology Co., Ltd., Shenzhen, China).

#### 2.6.2 HPLC determination of adrenaline and noradrenaline in adrenal glands

For high-performance liquid chromatography (HPLC), adrenal glands were homogenized in DEPROT solution (50 µL per 1 mg of tissue) containing 0.1 N perchloric acid (HClO_4_) and 0.2% MgCl_2_, followed by sonication and centrifugation 18,000 rpm for 30 min at 4 °C. Supernatants (50 µL) were injected using the autosampler of a Dionex UltiMate 3000 HPLC system (Thermo Scientific, Sunnyvale, CA, United States), equipped with an Acclaim Polar Advantage II C18 column (4.6 × 150 mm, 5 μm; Thermo Scientific, Waltham, MA, United States). Instrument control and chromatographic data processing were performed using Chromeleon 7 software (Thermo Scientific).

The mobile phase consisted of 98% ammonium formate buffer (pH 3.6; Fisher Scientific, Cambridge, United Kingdom) and 2% methanol (J.T. Baker, Griesheim, Germany), delivered at a flow rate of 0.5 mL/min. Electrochemical detection was carried out at +850 mV, and the column temperature was maintained at 25 °C. Standard solutions of noradrenaline (DL-noradrenaline hydrochloride, Sigma-Aldrich) and adrenaline ((±)-adrenaline hydrochloride, Sigma-Aldrich) were prepared in DEPROT from a 1 mg/mL methanolic stock, at concentration ranging from 0.5 to 50 μg/mL.

#### 2.6.3 qRT-PCR analysis of the liver and colon

Total RNA was isolated from liver and colon tissues using TRIzol™ Reagent (Invitrogen, Thermo Fisher Scientific, United States), according to the manufacturer’s instructions. Briefly, approximately 80–100 mg of tissue was homogenized in 1 mL of TRIzol using a homogenizer and sonicated for 30 s. After incubation at room temperature for 5 min, 200 µL of chloroform was added, samples were vigorously vortexed, incubated for 3 min, and centrifuged at 12,000 × g for 15 min at 4 °C. The aqueous phase was transferred to a new RNase-free tube, mixed with 500 µL of isopropanol, incubated for 10 min, and centrifuged at 12,000 × g for 10 min at 4 °C. The RNA pellet was washed with 75% ethanol, air-dried, and dissolved in 50 µL of RNase-free water. RNA concentration and purity were measured using a Take3 Microvolume Plate and an Epoch Microplate Spectrophotometer (BioTek, United States), while RNA integrity was confirmed by agarose gel electrophoresis.

cDNA was synthesized from total RNA using the High-Capacity cDNA Reverse Transcription Kit (Applied Biosystems, Thermo Fisher Scientific, United States). RNA samples were diluted to 100 ng/μL with nuclease-free water. Each 20 µL reaction contained diluted RNA and 2× reverse transcription master mix, as recommended by the manufacturer. A no-reverse transcriptase control (–RT) was included to verify the absence of genomic DNA contamination. Reverse transcription was performed at 25 °C for 10 min, 37 °C for 120 min, and 85 °C for 5 min cDNA samples were stored at −20 °C until further analysis.

For quantitative PCR, cDNA samples were diluted to 10 ng/μL. Each 10 µL reaction contained 2 µL of diluted cDNA, 5 µL of SYBR™ Green PCR Master Mix (Thermo Fisher Scientific, United States), 0.3 µL of each primer (10 µM), and nuclease-free water. Two negative controls were included: −RT and a no-template control (NTC). Primer sequences for the target genes toll-like receptor 4 (TLR4), tumor necrosis factor alpha (TNF-α), interleukin-1 beta (IL-1β), interleukin-10 (IL-10), and nuclear factor erythroid 2-related factor 2 (Nrf2), as well as for the reference gene hypoxanthine–guanine phosphoribosyltransferase 1 (HPRT1), are listed in Table 2. Amplification was performed on a StepOnePlus™ Real-Time PCR System (Applied Biosystems) under the following cycling conditions: 95 °C for 10 min, followed by 40 cycles of 95 °C for 15 s and 60 °C for 60 s.

**TABLE 2:** Primer sequences used for RT-qPCR.

Target	Forward primer (5′–3′)	Reverse primer (5′–3′)
HPRT1	CTC​ATG​GAC​TGA​TTA​TGG​ACA​GGA​C	GCA​GGT​CAG​CAA​AGT​CAA​AGT​C
TLR4	ATC​ATC​AGC​GAA​GGC​TTC​CA	GCT​AAG​AGG​CAA​GGA​CAA​TTC
TNFα	CCC​CAC​TTT​CTC​TTT​CCC​T	CCC​AGA​GCC​ATA​ATT​CCC​TT
IL1β	AAA​CAG​CAA​TGG​TCG​GGA​CA	GTC​CTG​GGG​AAG​GCA​TTA​GG
IL10	CTG​CAT​TTT​GCT​GGC​ATT​CTA​C	CTGCTTGATGTGGGTCTG
Nrf2	GAC​TTG​GAA​TTG​CCA​CCG​C	CCT​GTT​GCT​CTT​GTT​GGA​CAC

Gene expression levels were quantified using the 2^ΔΔ^Ct method (Livak and Schmittgen, 2001). ΔCt was calculated as the difference between the Ct value of the target gene and the Ct value of HPRT1. ΔΔCt represented the difference between the ΔCt of each sample and the mean ΔCt of the reference group.

Quantitative PCR experiments were performed in accordance with the MIQE guidelines. All relevant experimental parameters are reported to ensure transparency and reproducibility.

#### 2.6.4 Western blot analysis of the liver and colon

Liver and colon tissues were homogenized in ice-cold RIPA buffer (50 mM Tris-HCl, pH 7.2–7.5; 150 mM NaCl; 1% NP-40; 0.1% SDS; 0.5% Triton X-100; 10 mM EDTA; 10 mM EGTA) supplemented with protease (SigmaFAST, 10×) and phosphatase inhibitors (sodium orthovanadate, 100×). Homogenates were sonicated for 30 s, incubated on ice for 10 min, and centrifuged at 10,000 × g for 20 min at 4 °C. Supernatants were aliquoted and stored at −80 °C. Protein concentrations were determined using the Lowry method (Lowry et al., 1951).

Equal amounts of protein (30 μg) were separated on 8%–15% SDS-PAGE gels and transferred onto PVDF membranes (Millipore Sigma, United States). Membranes were blocked for 1 h at room temperature in 5% non-fat dry milk (sc-2324, Santa Cruz Biotechnology, United States) in TBST (50 mM Tris-HCl, pH 7.4; 150 mM NaCl; 0.2% Tween-20). Primary antibodies were incubated overnight at 4 °C: high mobility group box 1 (HMGB1; 1:1,000, ab18256, Abcam, United Kingdom), nuclear factor kappa B (total NF-κB p65; 1:1,000, cs#8242, Cell Signaling, United States), Bcl-2 associated X (BAX; 1:1,000, cs#2772, Cell Signaling, United States), B-cell lymphoma 2 (Bcl-2, 1:500, ab196495, Abcam, United States), copper-zinc superoxide dismutase (SOD1; 1:2000, ab16831, Abcam, United States), manganese superoxide dismutase (SOD2; 1:1,000, ab13533, Abcam, United States), and catalase (CAT; 1:2000, ab16731, Abcam, United States). After washing, membranes were incubated for 1 h at room temperature with HRP-conjugated goat anti-rabbit secondary antibody (1:30,000, Abcam, United States).

Protein bands were visualized using Clarity Western ECL substrate (BioRad, United States) with a ChemiDoc MP Imaging System (BioRad, United States) and imaged after 5 min of incubation. Membranes were stripped according to the Abcam mild stripping protocol and re-probed with HRP-conjugated anti-β-actin antibody (1:30,000, Abcam, United States) as a loading control. Band intensity was quantified using Image Lab software (v6.1, Bio-Rad, United States).

#### 2.6.5 Measurement of oxidative stress biomarkers in the liver

Liver tissue was homogenized in ice-cold 25 mM sucrose/10 mM Tris-HCl buffer (pH 7.5; 1:10 w/v) using a handheld Ultra-Turrax homogenizer (1,500 rpm; 3 × 10 s, IKA-Werk, Staufen, Germany), sonicated (10 kHz; 3 × 10 s, Bandeline Sonopuls HD 2070, Berlin, Germany), and centrifuged at 37,000 × g for 90 min at 4 °C (Beckman, Brea, CA, US). Supernatants were stored at −80 °C until analysis. All spectrophotometric assays were performed on a Shimadzu UV-1900i spectrophotometer (Shimadzu, Kyoto, Japan) with enzyme activities measured using a temperature-controlled cuvette holder (37 °C).

Oxidative stress biomarkers were assessed as previously described (Đurašević et al., 2022). Total superoxide dismutase (SOD, EC 1.15.1.1) and SOD2-MnSOD activities were determined by the adrenaline auto-oxidation method at 480 nm after SOD1-CuZnSOD inhibition with KCN (Misra and Fridovich, 1972). One unit of SOD activity was defined as the amount of protein that inhibited epinephrine auto-oxidation by 50% at 26 °C, and results were expressed as U/g wet mass (w.m.). SOD1 activity was calculated as the difference between total SOD and SOD2 and expressed as U/g w.m. Catalase (CAT, EC 1.11.1.6) activity was assessed by monitoring H_2_O_2_ decomposition at 240 nm and expressed as µmol H_2_O_2_/min/g w.m. (Claiborne, 1985). Glutathione peroxidase (GSH-Px, EC 1.11.1.9) activity was determined by monitoring the oxidation of NADPH in the presence of t-butyl hydroperoxide (Tamura et al., 1982), and expressed as nmol NADPH/min/g w.m. Glutathione reductase (GR, EC 1.8.1.3) activity was evaluated by monitoring NADPH oxidation and expressed as nmol NADPH/min/g w.m. (Glatzle et al., 1974), while glutathione S-transferase (GST, EC 2.5.1.18) activity was determined using 1-chloro-2,4-dinitrobenzene (CDNB) as substrate, and expressed as nmol GSH/min/g w.m. (Habig et al., 1974). Total glutathione (GSH) was determined at 412 nm as GSH oxidation by 5,5′-dithiobis-(2-nitrobenzoic acid) (DTNB) and NADPH reduction in the presence of GR (Griffith, 1980) and expressed as nmol/g w.m. Sulfhydryl (SH) group concentration was also determined using DTNB at 412 nm and expressed as μmol/mg w.m. (Ellman, 1959). Lipid peroxidation (LPO) was estimated as malondialdehyde (MDA) content using the thiobarbituric acid reactive substances (TBARS) assay and expressed as nmol MDA/mg w.m. (Rehncrona et al., 1980). Protein carbonyls (PCO) were measured using 2,4-dinitrophenylhydrazine (DNPH) by measuring hydrazone derivatives at 450 nm and expressed as nmol/mg protein (Mesquita et al., 2014).

#### 2.6.6 Statistical analysis

Statistical analysis was performed in R (v4.5.2). Outliers were detected using Dixon’s test. Data normality was tested using the Shapiro–Wilk test, while variance homogeneity was assessed using the F-test (SHAM vs. S) or Levene’s test (S, SM, SF, SMF). Parametric tests were applied to data with normal distribution and equal variances; otherwise, non-parametric or variance-adjusted tests were used.

Comparisons between SHAM and S groups were performed using Student’s t-test, Welch’s t-test (for unequal variances), or the Wilcoxon Rank Sum test (for non-normal data). Differences among S, SM, SF, and SMF groups were assessed using one-way ANOVA, Kruskal-Wallis ANOVA (non-normal data), or Welch’s ANOVA (heterogeneous variances). When significance was detected, *post hoc* tests included Tukey’s test (after one-way ANOVA), Dunn–Bonferroni (after Kruskal-Wallis), or Games-Howell (after Welch’s ANOVA). Statistical significance was set at p < 0.05. Results are presented as mean ± SEM. Effect sizes were calculated as Cohen’s d for two-group comparisons and η^2^ for ANOVA-based analyses. Detailed statistical outputs, including p-values, effect size estimates, and confidence intervals where applicable, are provided in Supplementary Table S3.

## 3 Results

### 3.1 Effects of meldonium and forskolin pretreatment on survival following CLP-induced sepsis

**FIGURE 2:**
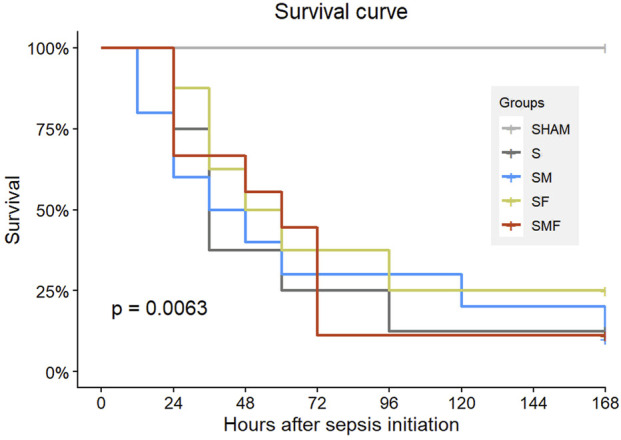
Effects of meldonium, forskolin, and their combination on survival of rats with CLP-induced sepsis. Kaplan–Meier survival curves showing 7-day survival of SHAM, septic (S), meldonium-pretreated septic (SM), forskolin-pretreated septic (SF), and combined meldonium + forskolin–pretreated septic (SMF) groups following CLP-induced polymicrobial sepsis. Statistical differences among groups were assessed using the log-rank (Mantel–Cox) test.

Kaplan–Meier survival analysis revealed a statistically significant difference in 7-day survival among the experimental groups (Figure 2; log-rank test). Pairwise comparisons showed that all CLP-induced septic groups (S, SM, SF, and SMF) had significantly reduced survival compared with the SHAM group. In contrast, no significant differences in survival were detected among the septic groups. Across septic groups, mortality occurred predominantly within the first 72 h after CLP, accounting for 75% (6/8), 70% (7/10), 62.5% (5/8), and 88.9% (8/9) of deaths in the S, SM, SF, and SMF groups, respectively. Notably, within the first 24 h, deaths were observed exclusively in the meldonium-pretreated septic group (SM).

### 3.2 Effects of meldonium and forskolin pretreatment on adrenal catecholamine content and serum markers of liver function in septic rats

In the adrenal glands, noradrenaline and adrenaline levels were significantly reduced in the septic (S) group compared with the SHAM group (p < 0.05) (Table 3). None of the pharmacological pretreatments (SM, SF, or SMF) restored catecholamine concentrations to control values. Total protein and albumin concentrations did not differ significantly between groups. Bilirubin levels were significantly increased in the S group compared with SHAM (p < 0.05), and this increase was not altered by any of the pretreatments. Among liver-associated enzymes, ALT activity was significantly elevated in the S group relative to SHAM (p < 0.05), AST, GGT, and LDH showed no statistically significant differences despite modest numerical differences among septic subgroups. Notably, ALP activity was significantly higher in the SF group compared with the untreated septic group (S) (p < 0.05).

**TABLE 3:** Adrenal catecholamine content and serum markers of liver function 24 h after CLP-induced sepsis.

Parameters	Groups				
	SHAM	S	SM	SF	SMF
Adrenal glands					
Noradrenaline (μg/mL)	3.5 ± 0.6	1.8 ± 0.2^a^	1.9 ± 0.2	1.8 ± 0.3	2.3 ± 0.3
Adrenaline (μg/mL)	25.2 ± 4.4	13.8 ± 1.8^a^	9.7 ± 2.0	10.8 ± 2.5	14.3 ± 2.2
Serum					
Total proteins (g/L)	60.0 ± 2.4	55.8 ± 2.0	52.7 ± 2.1	57.6 ± 2.0	54.8 ± 1.8
Albumin (g/L)	24.6 ± 1.1	23.4 ± 1.1	22.2 ± 2.1	24.7 ± 0.7	23.0 ± 0.7
Bilirubin (μmol/L)	1.5 ± 0.1	2.4 ± 0.4^a^	3.5 ± 0.8	3.3 ± 0.6	2.2 ± 0.4
ALP (U/l)	182.4 ± 18.5	181 ± 6.3	213.0 ± 15.6	226.4 ± 13.3^b^	208.2 ± 10.5
ALT (U/l)	95.9 ± 5.1	118.0 ± 11.9^a^	107.2 ± 10.6	138.5 ± 15.4	105.9 ± 10.5
AST (U/l)	446.4 ± 35.4	469.7 ± 42.6	514.7 ± 79.0	610.5 ± 54.1	477.3 ± 41.4
GGT (U/l)	3.0 ± 0.6	4.0 ± 0.9	5.8 ± 0.8	5.1 ± 1.1	4.1 ± 0.6
LDH (U/l)	2629.1 ± 228.6	2678.3 ± 133.7	2434.2 ± 260.5	2643.4 ± 160.9	2789.7 ± 164.0

Adrenal gland noradrenaline and adrenaline concentrations, and serum levels of total proteins, albumin, bilirubin, alkaline phosphatase (ALP), alanine aminotransferase (ALT), aspartate aminotransferase (AST), gamma-glutamyl transferase (GGT), and lactate dehydrogenase (LDH). All values are presented as mean ± SEM. a and b indicate p < 0.05 (a vs. SHAM, group; b vs. S group).

### 3.3 Gut microbiota composition before and after CLP-induced sepsis in rats treated with meldonium and forskolin

**FIGURE 3:**
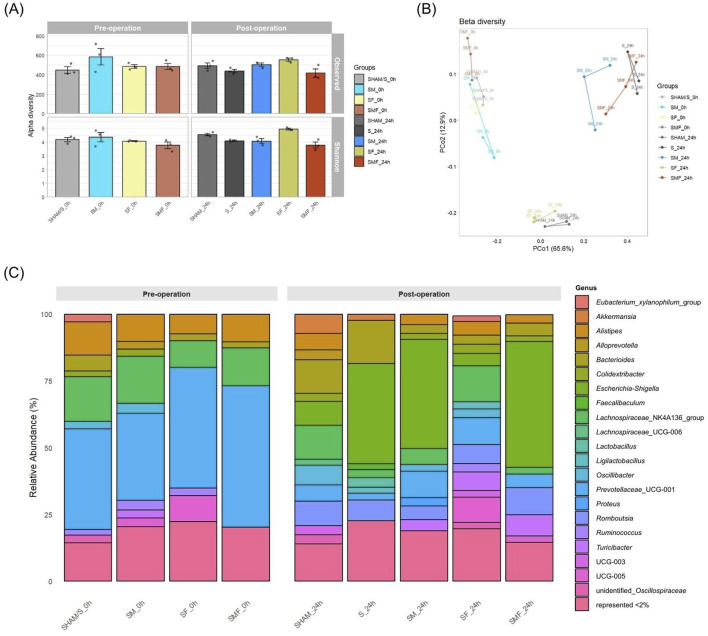
Global structure of gut microbiota before and after CLP-induced polymicrobial sepsis. **(A)** Alpha diversity of the gut microbiota assessed by Observed richness and Shannon diversity index at the pre-operative (0 h) and post-operative (24 h) time points. **(B)** Principal coordinates analysis (PCoA) based on Bray–Curtis distances showing clustering of samples according to sampling time and treatment. The first two axes explained 78.5% of total variance (PCo1 = 65.6%, PCo2 = 12.9%). **(C)** Relative abundance of dominant bacterial genera at the pre-operative (0 h) and post-operative (24 h) time points.

Gut microbiota analyses were based on cage-level composite samples, and findings reflect group-level microbial patterns rather than individual-animal variability. Alpha diversity, assessed using Observed richness and Shannon diversity, showed no significant differences between experimental groups at either the pre-operative or post-operative time point (Figure 3A). Beta diversity analysis based on Bray–Curtis distances revealed separation of samples primarily according to sampling time and treatment (Figure 3B), with the first two PCoA axes explaining 78.5% of the total variance. PERMANOVA analysis indicated that sampling time represented the dominant source of variation in microbial community composition (p = 0.001). Consistent with this, samples collected at 24 h clustered separately from their respective baseline (0 h) counterparts across all experimental groups, primarily along PCo1. In addition, separation of specific groups was observed along PCo2, indicating secondary axis–associated variation in community structure. Pairwise PERMANOVA comparisons between individual groups did not reach statistical significance (p > 0.05). At the genus level, pre-operative groups exhibited broadly similar microbial profiles, dominated by *Prevotellaceae*_UCG-001, *Oscillibacter*, and *Lachnospiraceae*-related taxa (Figure 3C). At 24 h after surgery, SHAM animals showed only modest compositional changes, whereas CLP-induced sepsis was associated with pronounced microbial restructuring, characterized by expansion of *Escherichia–Shigella* and reduction of several commensal taxa. Pharmacological pretreatments modified post-operative microbial profiles relative to untreated sepsis (Figure 3C).

**FIGURE 4:**
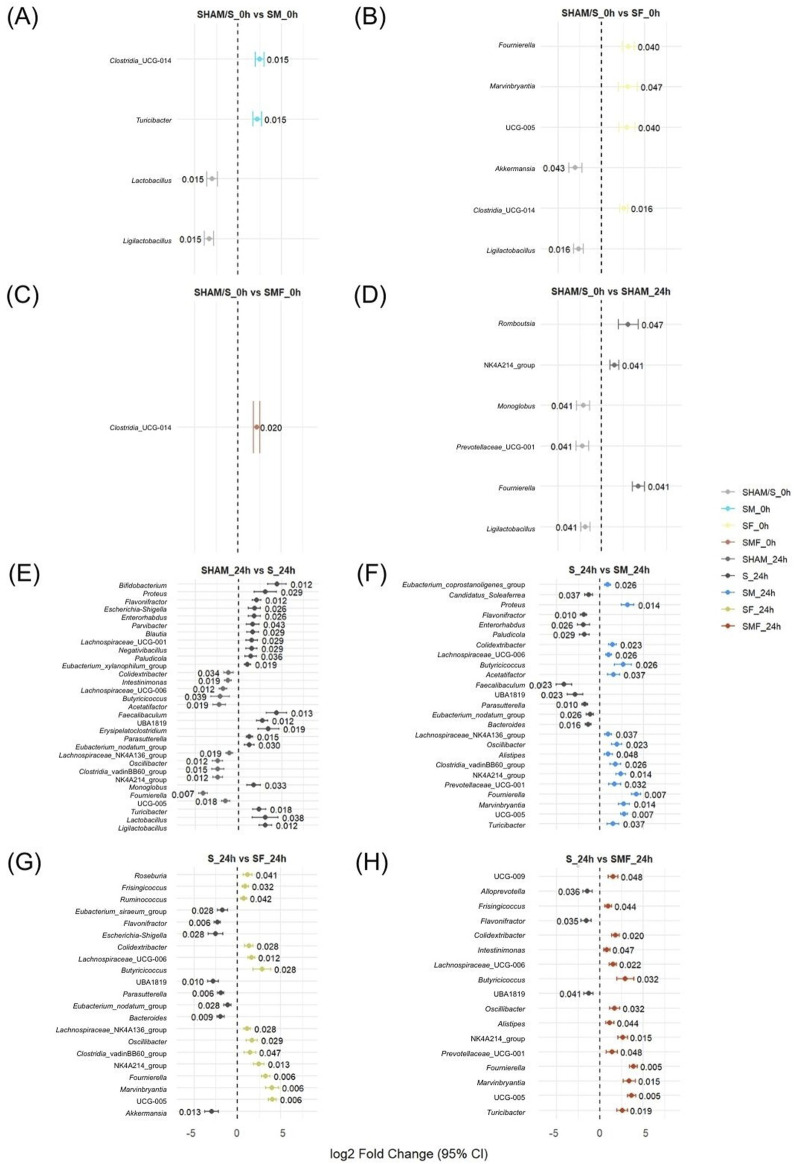
Differentially abundant gut bacterial taxa identified by ANCOM-BC analysis. Statistically significant taxa (p < 0.05) were identified across pairwise comparisons between pre-operative treatment groups (SM_0h, SF_0h, SMF_0h vs. SHAM/S_0h **(A–C)**), postoperative SHAM controls (SHAM_24h **(D,E)**), untreated CLP animals (S_24h** (E–H)**), and CLP animals pretreated with meldonium (SM_24h** (F)**), forskolin (SF_24h** (G)**), or their combination (SMF_24h** (H)**). Log2 fold changes with 95% confidence intervals are shown. Positive values indicate higher abundance in the comparison group relative to the reference group, while negative values indicate lower abundance. Numbers displayed next to each point indicate the associated p-values. Only taxa reaching ANCOM-BC significance are displayed.

Differential abundance analysis (ANCOM-BC) revealed significant (p < 0.05) pretreatment- and surgery-associated shifts in gut microbial composition across both pre-operative and post-operative comparisons (Figure 4). At baseline (0 h), metabolic pretreatments induced distinct microbiota signatures (Figures 4A–C). Meldonium pretreatment (SM_0h) was associated with shifts in several bacterial taxa, including an increase in *Clostridia*_UCG-014 and a reduction in *Lactobacillus* (Figure 4A). Forskolin pretreatment (SF_0h) induced distinct baseline alterations, characterized by enrichment of selected anaerobic taxa and reduction of mucosa-associated genera (Figure 4B). The combined pretreatment (SMF_0h) produced a more restricted baseline effect, with a significant increase limited to *Clostridia*_UCG-014 (Figure 4C). Comparison of SHAM/S_0h and SHAM_24h revealed surgery-associated microbial shifts, including increased *Fournierella* and decreased *Ligilactobacillus* (Figure 4D). Direct comparison between SHAM_24h and S_24h demonstrated an expansion of opportunistic taxa, including *Escherichia–Shigella*, and a reduction of several commensal genera (Figure 4E). Among pretreated septic animals, each intervention produced a distinct microbial response relative to untreated sepsis (Figures 4F–H). Meldonium pretreatment (SM_24h) was associated with increased abundance of *Lachnospiraceae*-related taxa and reduced abundance of several opportunistic genera (Figure 4F). Forskolin pretreatment (SF_24h) increased selected anaerobic genera while reducing *Escherichia–Shigella* (Figure 4G). Combined pretreatment (SMF_24h) resulted in enrichment of multiple commensal taxa relative to untreated sepsis (Figure 4H).

### 3.4 Effects of meldonium and forskolin pretreatment on histopathological alterations in the ileum and colon after CLP-induced sepsis

**FIGURE 5:**
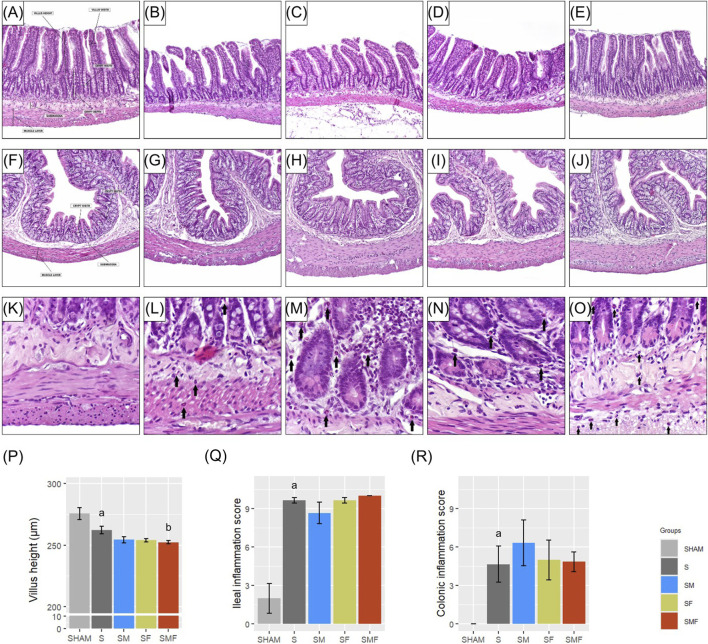
Histopathological alterations in the ileum and colon 24 h after CLP-induced polymicrobial sepsis. Representative hematoxylin and eosin (H&E) stained sections of the ileum **(A–E)** and colon **(F–J)** are shown (original magnification ×100). Ileum: **(A)** SHAM – preserved villus architecture and intact epithelial lining; **(B)** S – shortened and blunted villi with inflammatory cell infiltration; **(C)** SM – villus shortening comparable to S; **(D)** SF – villus shortening similar to S; **(E)** SMF – most pronounced villus atrophy. Colon: **(F)** SHAM – preserved crypt architecture and normal mucosal organization; **(G)** S, **(H)** SM, **(I)** SF, **(J)** SMF – preserved crypt structure with inflammatory cell infiltration. Higher magnification images illustrate inflammatory infiltration **(K–O)**: **(K)** SHAM – absence of inflammation; **(L)** S, **(M)** SM, **(N)** SF, **(O)** SMF – marked inflammatory cell infiltration in the lamina propria, mucosa, and submucosa of both ileum and colon (arrows). Quantitative morphometric and inflammatory analyses are shown for ileal villus height **(P)**, ileal inflammation score **(Q)**, and colonic inflammation score **(R)**. Data are presented as mean ± SEM. a and b indicate p < 0.05 (a vs. SHAM group; b vs. S group).

Histopathological analysis revealed marked intestinal vulnerability during early sepsis (Figure 5). In the ileum, villus height was significantly reduced in the septic group (S) compared with SHAM controls (p < 0.05) (Figure 5P). None of the applied pretreatments (SM, SF, or SMF) prevented villus shortening. Moreover, villus height was further reduced in the SMF group compared with untreated septic animals (p < 0.05), indicating exacerbation of structural injury. Villus width, crypt parameters, and intestinal wall layer thickness remained unchanged across groups. Inflammation scores in the ileum were markedly increased in all CLP-induced groups compared with SHAM (p < 0.05), with no reduction observed in any pretreatment group (Figure 5Q). In the colon, no statistically significant changes were detected in crypt depth, crypt width, mucosal thickness, or muscular layer thickness across groups. However, colonic inflammation was increased in all CLP-induced groups compared with SHAM, with no attenuation by pretreatments (Figure 5R). Detailed quantitative data for all measured intestinal parameters are provided in the Supplementary Table S1.

### 3.5 Effects of meldonium and forskolin pretreatment on transcriptional expression of inflammatory mediators in the colon and liver after CLP-induced sepsis

**FIGURE 6:**
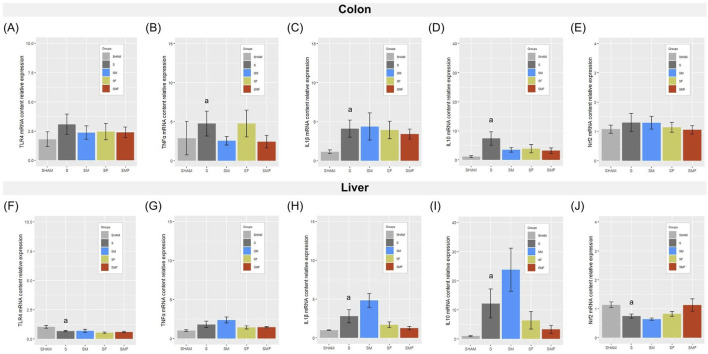
Expression of inflammatory mediators in the colon and liver 24 h after CLP-induced sepsis. Relative mRNA expression levels of TLR4 **(A,F)**, TNFα **(B,G)**, IL1β **(C,H)**, IL10 **(D,I)**, and Nrf2 **(E,J)** were determined in the colon **(A–E)** and liver **(F–J)** of SHAM, septic (S), meldonium-pretreated septic (SM), forskolin-pretreated septic (SF), and combined meldonium + forskolin-pretreated septic (SMF) rats 24 h after CLP-induced polymicrobial sepsis. Gene expression levels were normalized to HPRT1 and expressed relative to the SHAM group. Data are presented as mean ± SEM. a and b indicate p < 0.05 (a vs. SHAM group; b vs. S group).

In the colon, CLP-induced sepsis triggered a pronounced pro-inflammatory transcriptional response (Figures 6A–E), with significantly increased TNFα, IL1β, and IL10 mRNA expression in the septic group compared with SHAM controls (p < 0.05). None of the pharmacological pretreatments significantly modified cytokine expression, while colonic TLR4 and Nrf2 mRNA levels remained unchanged across all groups. In the liver, sepsis was associated with significantly increased IL1β and IL10 mRNA expression and reduced TLR4 and Nrf2 mRNA expression in untreated septic animals compared with SHAM controls (p < 0.05) (Figures 6F–J). These alterations were not affected by any of the pretreatments, whereas hepatic TNFα mRNA expression did not differ significantly among experimental groups.

### 3.6 Effects of meldonium and forskolin pretreatment on total NF-κB p65, HMGB1, and BAX/Bcl-2 protein content in the colon and liver after CLP-induced sepsis

**FIGURE 7:**
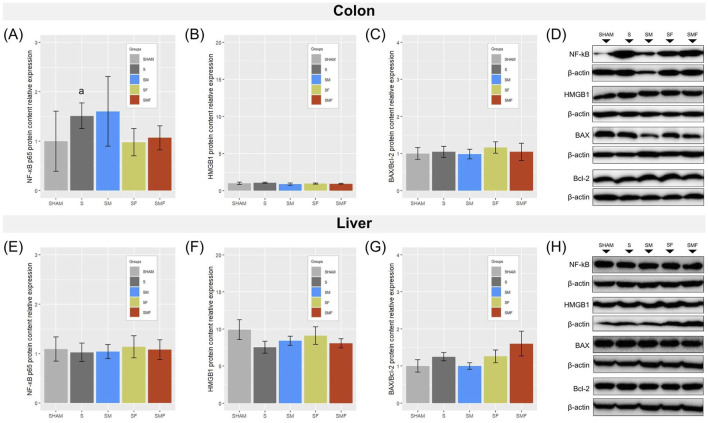
Total NF-κB p65, HMGB1, and BAX/Bcl-2 in the colon and liver 24 h after CLP-induced sepsis. Relative protein expression of total NF-κB p65 **(A,E)**, HMGB1 **(B,F)**, and the BAX/Bcl-2 ratio **(C,G)** was analyzed in the colon **(A–D)** and liver **(E–H)** of SHAM, septic (S), meldonium-pretreated septic (SM), forskolin-pretreated septic (SF), and combined meldonium + forskolin-pretreated septic (SMF) rats 24 h after CLP-induced polymicrobial sepsis. Representative Western blot images are shown **(D,H)**. Protein levels were normalized to β-actin and expressed relative to SHAM. Data are presented as mean ± SEM. a and b indicate p < 0.05 (a vs. SHAM group; b vs. S group).

In the colon, CLP-induced sepsis led to a significant increase in total NF-κB p65 protein expression compared with SHAM animals (p < 0.05), while no significant differences were observed among the septic groups that received pharmacological pretreatments (Figures 7A,D). Colonic HMGB1 protein levels and the BAX/Bcl-2 ratio did not differ significantly between experimental groups (Figures 7B–D). Hepatic protein expression of total NF-κB p65, HMGB1, and the BAX/Bcl-2 ratio remained unchanged across all experimental groups (Figures 7E–H).

### 3.7 Effects of meldonium and forskolin pretreatment on hepatic oxidative stress biomarkers after CLP-induced sepsis

**FIGURE 8:**
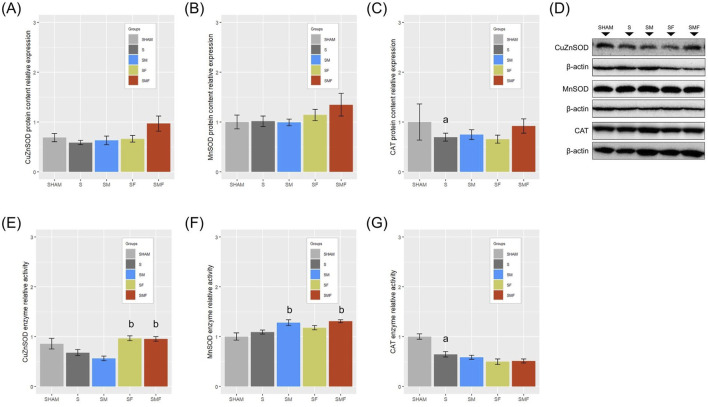
Antioxidant enzyme expression and activity in the liver 24 h after CLP-induced sepsis. Protein expression and enzymatic activity (U/g w.m.) of hepatic antioxidant enzymes SOD1 **(A,E)**, SOD2 **(B,F)**, and CAT **(C,G)** in SHAM, septic (S), meldonium-pretreated septic (SM), forskolin-pretreated septic (SF), and combined meldonium + forskolin-pretreated septic (SMF) rats 24 h after CLP. Representative Western blots are shown **(D)**. Protein levels were normalized to β-actin. Data are expressed relative to SHAM and presented as mean ± SEM. a and b indicate p < 0.05 (a vs. SHAM group; b vs. S group).

Hepatic protein expression of SOD1 and SOD2 did not differ significantly among experimental groups (Figures 8A,B,D); however, enzymatic activity analysis revealed pretreatment-specific modulation. SOD1 activity was significantly increased in the SF and SMF groups, while SOD2 activity was elevated in the SM and SMF groups compared with untreated sepsis (Figures 8E,F). CAT protein expression and activity were significantly reduced in the untreated septic group compared with SHAM animals (p < 0.05), with no significant differences observed in pretreated groups (Figures 8C,D,G).

**FIGURE 9:**
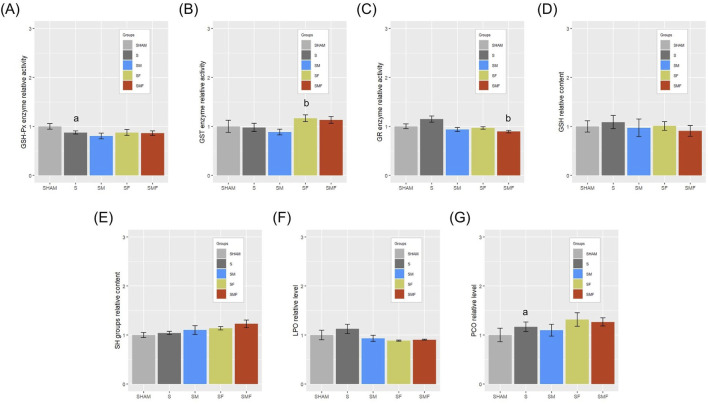
Glutathione-dependent enzymes and oxidative stress markers in the liver 24 h after CLP-induced sepsis. Hepatic activities (U/g w.m.) of GSH-Px **(A)**, GST **(B)**, and GR **(C)**, along with levels of GSH (**(D)** nmol NADPH/min/g w.m.), SH groups (**(E)** μmol/mg w.m.), LPO (**(F)**, nmol MDA/mg w.m.), and PCO (**(G)**, nmol/mg protein) were measured in SHAM, septic (S), meldonium-pretreated septic (SM), forskolin-pretreated septic (SF), and combined meldonium + forskolin-pretreated septic (SMF) rats 24 h after CLP. Data are expressed relative to SHAM and presented as mean ± SEM. a and b indicate p < 0.05 (a vs. SHAM group; b vs. S group).

CLP-induced sepsis was associated with a significant reduction in GSH-Px activity in the untreated septic group compared with SHAM animals, with no significant differences observed in pretreated groups (Figure 9A). GST activity was significantly increased in the SF group, whereas GR activity was reduced in the SMF group relative to untreated sepsis (Figures 9B,C). Levels of GSH, total SH groups, and LPO did not differ significantly among experimental groups (Figures 9D–F). In contrast, PCO content was significantly increased in septic animals compared with SHAM controls, with no significant differences detected among the pretreated groups (Figure 9G). Absolute values of antioxidant enzyme activities and oxidative stress markers are provided in Supplementary Table S2.

## 4 Discussion

Sepsis is a rapidly evolving systemic disorder in which early host responses critically shape downstream outcomes, including organ dysfunction and mortality (Husabø et al., 2020; Srdić et al., 2024). In this study, pre-septic pharmacological modulation of host metabolism and microbiota did not lead to improved survival, reduced early intestinal injury, or consistent suppression of early inflammatory signaling during the first 24 h after CLP. Importantly, the lack of statistically significant functional efficacy represents a key finding, highlighting the limits of preconditioning in modulating host responses during early polymicrobial sepsis. Importantly, the absence of functional improvement in this severe CLP-induced sepsis model highlights that the earliest phase of polymicrobial sepsis is dominated by fast host-intrinsic pathophysiological responses, which can override the effects of pharmacological modulation (Buras et al., 2005).

### 4.1 Early adrenal exhaustion and survival of septic rats

A hallmark of early sepsis is dysregulation of the hypothalamic-pituitary-adrenal (HPA) axis and catecholamine metabolism (Kanczkowski et al., 2016). In line with this, septic animals in the present study showed marked depletion of adrenal noradrenaline and adrenaline, consistent with sustained sympathetic activation and rapid catecholamine consumption (Kanczkowski et al., 2015). Consistent with our previous findings, comparable impairments in catecholamine availability during sepsis have been reported in our earlier experimental models, including fecal-induced peritonitis and LPS-induced endotoxemia (Đurašević et al., 2021a; Đurašević et al., 2022). Notably, none of the pharmacological pretreatments attenuated the reduction in adrenal catecholamine content, indicating that meldonium and forskolin did not prevent early neuroendocrine exhaustion. Given the essential role of catecholamines in maintaining hemodynamic stability and coordinating the systemic stress response, their early depletion likely contributes to impaired host adaptation and increased vulnerability during the initial phase of sepsis (Kanczkowski et al., 2015; Kanczkowski et al., 2016).

Notably, early mortality within the first 24 h after CLP occurred exclusively in meldonium-pretreated septic animals, while all other septic groups survived this critical initial phase. Although 7-day survival did not differ significantly among pretreated septic groups, this early divergence suggests that key determinants of outcome are established during the earliest stage of sepsis. Additionally, certain patterns observed in this study were consistent with a trade-off of preconditioning under severe stress, including early deaths limited to meldonium-pretreated animals and aggravated ileal villus shortening in the combined pretreatment group, suggesting that metabolic constraints may increase vulnerability during the earliest septic phase. Our previous studies have indicated that meldonium may unfavorably affect survival under conditions of severe energetic stress, with increased mortality reported in fecal-induced peritonitis and selective mortality observed in LPS-induced endotoxemia (Đurašević et al., 2021a; 2022). Mechanistically, inhibition of carnitine-dependent fatty acid utilization has been proposed to aggravate energetic instability during severe infection. In septic stress, where multiple systemic disturbances already constrain adaptive metabolic responses and energetic demand is markedly increased, substrate utilization must rapidly adapt to sustain organ function. Under such conditions, preconditioning that restricts metabolic flexibility may further exacerbate energetic imbalance and limit the capacity to compensate for acute systemic stress. Within this framework, early deaths restricted to meldonium-pretreated animals may reflect impaired metabolic adaptability during the most vulnerable phase of sepsis. These observations should be considered hypothesis-generating and require confirmation in adequately powered survival-focused studies.

### 4.2 Early intestinal damage and downstream gut-liver axis alterations

Histopathological analysis demonstrated pronounced intestinal vulnerability during early sepsis, manifested by significant ileal villus shortening. This alteration is consistent with previous reports showing that CLP-induced sepsis rapidly induces villus atrophy, detectable within the first few hours after sepsis onset (Meng et al., 2019; Peng et al., 2023). In contrast, colonic architecture remained largely preserved, indicating a segment-specific pattern of intestinal injury during early sepsis, with preferential involvement of the ileum. None of the applied pretreatments prevented villus shortening, and combined pretreatment with meldonium and forskolin further exacerbated ileal injury in septic animals. This deterioration may reflect treatment-related metabolic vulnerability of the intestinal epithelium, whereby reduced energy availability and altered cAMP signaling increase susceptibility of differentiated villus epithelial cells to early damage. In parallel, extensive inflammatory infiltration was observed throughout the intestinal wall in all septic groups, confirming that structural injury and inflammation develop concurrently during early sepsis (Peng et al., 2023). Notably, preserved crypt depth and mucosal thickness suggest that proliferative stem cell compartments remain largely intact within the first 24 h after CLP, despite substantial injury to the differentiated villus epithelium (Williams et al., 2015).

The colon is a critical component of the intestinal barrier, and sepsis-induced disruption of epithelial integrity has been associated with increased intestinal permeability and bacterial translocation, promoting local and systemic inflammatory responses (Yoseph et al., 2016; Cao et al., 2021). In our experiment, colonic architecture remained largely preserved despite evident inflammatory cell infiltration and robust inflammatory activation at the cytokine level. Specifically, analysis of early colonic responses revealed transcriptional induction of TNFα, IL1β, and IL10, consistent with the initial pro-inflammatory phase accompanied by an early compensatory anti-inflammatory component described in experimental sepsis (Tamayo et al., 2011; Chao et al., 2020). This cytokine profile was not significantly modified by pharmacological pretreatments, indicating a limited capacity of meldonium and forskolin to influence early colonic inflammatory transcription. Notably, this cytokine induction occurred in the absence of detectable changes in TLR4 transcript levels, indicating that early inflammatory activation was not accompanied by transcriptional upregulation of this receptor. Similarly, unchanged Nrf2 expression indicates that antioxidant transcriptional programs in the colon are not yet engaged at this early time point. At the protein level, sepsis was associated with a selective increase in total NF-κB p65, in line with previous reports describing early changes in NF-κB pathway components in the intestinal epithelium during the acute phase of CLP-induced sepsis (Cao et al., 2021). In contrast, HMGB1 content and the BAX/Bcl-2 ratio remained unchanged, suggesting cytokine-driven inflammatory activation without overt shifts in the assessed apoptotic balance at this time point. This pattern aligns with evidence indicating that HMGB1 involvement in sepsis is typically linked to later-stage inflammatory amplification and apoptotic signaling, while early inflammatory responses may proceed independently of HMGB1 modulation (Qin et al., 2006). Accordingly, pronounced apoptotic signaling has been reported predominantly in metabolically active organs, with limited evidence in colonic tissue during the early phase after CLP (Messaris et al., 2007; Abdelnaser et al., 2023). Although meldonium and forskolin have demonstrated anti-inflammatory and anti-apoptotic effects in other experimental contexts (El-Agroudy et al., 2016; Guo et al., 2018; Đurašević et al., 2022), such effects were not evident in the colon under the present experimental conditions. However, it should be noted that apoptosis was assessed only through the BAX/Bcl-2 ratio, while other markers of apoptotic processes or alternative regulated cell death pathways were not evaluated.

In the context of early sepsis, intestinal injury is recognized as a key interface linking gut-derived inflammatory signals with hepatic dysfunction via the gut-liver axis (Sun et al., 2020). Within this framework, the liver acts as a central regulator of systemic metabolic and immune responses and is among the first organs affected during sepsis, where gut-derived inflammatory signals and microbial products contribute to the development of sepsis-induced liver injury (Guo et al., 2025). Under the present experimental conditions, early sepsis was associated with a selective hepatic inflammatory response, characterized by increased IL1β and IL10 mRNA expression, while TNFα transcript levels remained unchanged, indicating the absence of generalized activation of cytokine transcription within the first 24 h after CLP. In contrast to the colon, hepatic TLR4 mRNA expression was significantly reduced, indicating an early, tissue-specific alteration of innate immune receptor regulation during sepsis. At the protein level, hepatic total NF-κB p65, HMGB1, and the BAX/Bcl-2 ratio remained unchanged, indicating no detectable alterations in the assessed inflammatory and apoptotic markers during the early phase of sepsis. Despite previously reported tissue- and model-dependent effects of meldonium and forskolin on inflammatory and apoptotic pathways (Guo et al., 2018; Đurašević et al., 2019; Đurašević et al., 2021b; Xiaohua et al., 2019), none of the applied pretreatments significantly modified these hepatic markers. As apoptosis was evaluated only through the BAX/Bcl-2 ratio, involvement of additional apoptotic mechanisms or alternative regulated cell death pathways cannot be excluded. In contrast, the hepatic response to early sepsis was dominated by disturbances in redox homeostasis. Reduced Nrf2 mRNA expression, accompanied by decreased CAT protein expression and enzymatic activity, reduced GSH-Px activity, and increased PCO, collectively indicate early oxidative protein damage. This pattern is consistent with previous observations showing that PCO is an early marker of oxidative stress in sepsis and may occur independently of LPO (Galley, 2010; Prauchner, 2017). Accordingly, unchanged LPO in the present study supports the concept that oxidative protein modification constitutes an early redox disturbance in hepatic tissue during sepsis. Although pharmacological pretreatments exerted selective, enzyme-specific effects within the antioxidant system, they did not significantly prevent the main sepsis-associated redox alterations. This suggest that under the present experimental conditions, partial modulation of antioxidant pathways alone is insufficient to counteract early hepatic vulnerability. Furthermore, all CLP-induced septic animals exhibited clear signs of early hepatocellular stress and cholestatic dysfunction, as reflected by elevated serum bilirubin and ALT levels. Importantly, elevated serum bilirubin has been identified as an independent predictor of mortality in septic and critically ill patients, underscoring the clinical relevance of early cholestatic disturbances and their association with adverse outcomes (Peng et al., 2021; Yang et al., 2021). In contrast to these general septic effects, ALP levels increased exclusively in forskolin-treated septic animals, suggesting a selective influence of forskolin on hepatic functional parameters. This observation is consistent with previous reports describing increased AST, ALT, and ALP following *Coleus forskohlii* administration in healthy mice (Virgona et al., 2013). These findings raise the possibility that cAMP-driven signaling may contribute to hepatic enzyme modulation during systemic stress such as sepsis. However, alternative explanations, including additive pharmacological effects or altered hepatic susceptibility, cannot be excluded and warrant further investigation.

### 4.3 Gut microbiota remodeling during early sepsis does not translate into organ protection

Intestinal structural integrity and gut microbiota composition are closely interdependent during sepsis, as epithelial injury and barrier dysfunction are accompanied by marked alterations in gut microbial communities (Pan et al., 2022; Peng et al., 2023). In line with this, our results demonstrated pronounced restructuring of the gut microbiota following CLP-induced sepsis, with beta diversity primarily driven by sampling time rather than pharmacological pretreatment, while alpha diversity remained preserved. This pattern is consistent with experimental and clinical studies showing that early sepsis induces rapid compositional shifts in the gut microbiota that precede a global loss of richness or evenness (Barlow et al., 2022; de Vos et al., 2022). Such early dysbiosis therefore reflects functional destabilization of the microbial ecosystem rather than immediate microbial depletion. Given the cage-level sampling strategy, microbiota data should be interpreted as group-level shifts rather than individual causal determinants of outcome. Consequently, individual-level associations between microbiota composition and host response parameters could not be assessed within the current design.

Importantly, metabolic pretreatments exerted distinct baseline effects on gut microbial composition, indicating that host metabolic conditioning can modify the pre-septic microbial state. Similar pretreatment-associated shifts in microbiota composition have been reported in experimental models, supporting the concept that host metabolic and physiological status is a determinant of microbial community structure (de Vos et al., 2022; Zhao et al., 2023). Meldonium pretreatment induced selective shifts in metabolically responsive taxa, whereas forskolin produced broader baseline alterations, including enrichment of fermentative and short-chain fatty acid (SCFA)-associated genera alongside a reduction in *Akkermansia*, a mucin-associated taxon implicated in epithelial integrity and metabolic regulation (Reunanen et al., 2015). Notably, the combined pretreatment resulted in the most limited baseline alterations, suggesting a balancing interaction between the two compounds.

Following CLP, sepsis induced a characteristic dysbiotic signature marked by expansion of opportunistic and inflammation-associated taxa, including *Proteus*, *Flavonifractor*, *Escherichia–Shigella*, and *Bifidobacterium*, together with depletion of multiple beneficial commensals and SCFA-producing genera such as *Butyricicoccus*, *Intestinimonas*, *Oscillibacter*, *Lachnospiraceae*_UCG-006, and *Acetatifactor*. This direction of microbial change closely aligns with previous reports describing sepsis-associated dysbiosis as a shift toward facultative pathobionts and concomitant loss of SCFA-producing bacteria, collectively compromising epithelial support and anti-inflammatory microbial functions (Pan et al., 2022; Peng et al., 2023; Zhao et al., 2023). Expansion of *Proteobacteria*-related taxa such as *Escherichia–Shigella* and *Proteus* is widely recognized as a microbial signature of epithelial stress and inflammatory environments during sepsis (Barlow et al., 2022; de Vos et al., 2022).

Pharmacological pretreatments differentially shaped the septic microbiota. Meldonium pretreatment induced a heterogeneous response characterized by enrichment of several beneficial taxa, including *Butyricicoccus*, *Oscillibacter*, *Alistipes*, and Prevotellaceae_UCG-001, alongside persistence of selected dysbiosis-associated genera such as *Proteus* and *Clostridia*_vadinBB60_group. Forskolin pretreatment promoted enrichment of fermentative and SCFA-associated genera (*Roseburia*, *Ruminococcus*, *Butyricicoccus*, *Oscillibacter*) while reducing several sepsis-associated taxa, including *Escherichia–Shigella* and *Flavonifractor*, but was also associated with reduced *Akkermansia*. The combined meldonium-forskolin pretreatment elicited the most coherent microbial profile, characterized by enrichment of multiple commensal taxa (*Butyricicoccus*, *Intestinimonas*, *Oscillibacter*, *Alistipes*, *Prevotellaceae*_UCG-001, *Turicibacter*) and attenuation of selected dysbiosis-associated signatures (Louis and Flint, 2017). Despite partial restoration of commensal and SCFA-associated taxa, microbiota modulation did not result in attenuation of intestinal inflammation or villus shortening. Notably, the combined pretreatment group exhibited pronounced microbial recovery alongside aggravated ileal structural damage, indicating that microbiota normalization appears insufficient to prevent early sepsis-associated epithelial injury. Collectively, these findings suggest a dominant role of host-driven mechanisms, rather than microbial composition *per se*, in shaping intestinal pathology during the earliest phase of sepsis.

Taken together, these findings highlight the first 24 h after CLP as a critical window during which coordinated but compartment-specific host responses shape the early trajectory of sepsis. In this phase, adrenal catecholamine depletion reflects rapid neuroendocrine exhaustion, while the intestine emerges as an early site of vulnerability characterized by ileal structural injury, colonic inflammatory activation, and pronounced microbiota restructuring. Although pharmacological pretreatments partially restored commensal and SCFA-associated taxa at the group level, these microbial shifts were not accompanied by measurable improvement in intestinal architecture or early inflammatory signaling. In parallel, the liver exhibited a distinct response marked by selective cytokine transcription and predominant redox imbalance, rather than detectable alterations in the assessed inflammatory or apoptotic markers. Collectively, these patterns indicate that early septic pathology appears to be governed predominantly by rapid, host-intrinsic stress programs that constrain the capacity of pre-septic metabolic modulation to influence early septic trajectories. In this framework, the first 24 h after sepsis onset may represent a period of limited pharmacological plasticity, during which classical metabolic and anti-inflammatory modulation is insufficient to override systemic stress-driven injury. Profound physiological disturbances characterizing early sepsis, including systemic metabolic imbalance, redox dysregulation, and impaired tissue perfusion, may alter drug pharmacodynamics and intracellular signaling responsiveness. Under such conditions, expected pathway-targeted effects may not translate into effective target engagement or measurable tissue protection. This possibility may partly explain the limited functional impact observed in the present study. Future studies should therefore directly assess pathway engagement and drug exposure during early sepsis to clarify the basis of this limited efficacy.

## 5 Conclusion

This study indicates that the earliest phase of polymicrobial sepsis is characterized by early gut-liver injury accompanied by marked microbiota dysbiosis, hepatic oxidative imbalance, and adrenal catecholamine depletion. These alterations emerge within the first 24 h after sepsis induction and precede overt organ failure and mortality. Although gut microbiota composition and selected antioxidant pathways were modified before sepsis onset, these changes did not translate into measurable protection against early intestinal injury, adrenal dysfunction, or reduced survival at the group level. Together, these findings support the concept that the earliest septic phase represents a state of limited pharmacological plasticity, dominated by rapid host-driven stress responses that constrain the impact of metabolic and microbiota-oriented modulation. While smaller treatment effects cannot be fully excluded within the constraints of the severe CLP model, the specific microbiota alterations and metabolic preconditioning examined here appear insufficient to determine early gut-liver injury or disease trajectory, highlighting the first 24 h as a critical window largely dominated by host-driven mechanisms. Taken together, our results support the need to reconsider pharmacological strategies in early sepsis by increasing attention to host-driven pathophysiological processes and by defining the temporal boundaries of pharmacological responsiveness through direct verification of target engagement under severe septic stress conditions.

## 6 Limitations

While this study provides novel insight into early host-microbiota-organ interactions during sepsis, several limitations should be considered. Although treatment doses were selected based on prior studies demonstrating metabolic pathway modulation, direct verification of target engagement was not performed. Additionally, the forskolin preparation consisted of a *Coleus forskohlii* extract containing 10% active compound; therefore, potential effects of other constituents cannot be fully excluded. All analyses were performed at a single early time point (24 h after CLP), which limits insight into the temporal progression of inflammatory and microbiota-related changes. Although inclusion of additional time points would help distinguish transient from sustained alterations and better link early molecular events with later outcomes, such an approach is complicated by early mortality in the CLP model. Expanding longitudinal analyses would require substantially larger animal cohorts, raising ethical and practical considerations. Consequently, while the current design allowed detection of large effects, smaller or moderate differences across measured parameters among septic subgroups may have remained undetected. This consideration is particularly relevant when interpreting intergroup comparisons and evaluating the potential biological relevance of moderate changes that did not reach statistical significance. In addition, although histological evidence of intestinal injury was observed, functional assessments of gut barrier integrity were not performed, limiting conclusions about the physiological consequences of villus shortening, including potential functional barrier impairment or bacterial translocation. Moreover, gut microbiota analysis was based on cage-level composite samples, representing a methodological limitation of the present study. This approach reduces biological resolution and precludes individual-level association analyses between microbiota composition and host response parameters. Furthermore, inflammatory signaling analysis focused on selected cytokines and pathways, and broader transcriptomic or proteomic profiling could reveal additional mechanisms involved in early sepsis-induced tissue injury. Finally, although the severe CLP model is ideal for studying mechanisms underlying early organ injury and rapid deterioration, its severity restricts the direct translation of these findings to less severe or later phases of sepsis, in which host responses may be more responsive to pharmacological modulation.

## Data Availability

The original contributions presented in the study are publicly available. This data can be found in the NCBI Sequence Read Archive (SRA) under BioProject accession number PRJNA1451941.
