# Supplementary material for: Dissociation between gut microbiota remodeling and early gut-liver injury in CLP-induced sepsis
Source: Front Pharmacol. 2026 Apr 20;17:1800088. doi: 10.3389/fphar.2026.1800088 (PMC13136135; doi:10.3389/fphar.2026.1800088)
Supplement: Supplementary file 1 [file Table1.pdf]

Supplementary Material

1 Supplementary Tables

Supplementary Table S1. Quantitative histopathological parameters of ileum and colon 24 hours after CLP-induced sepsis. Ileal parameters included villus height, villus width, crypt depth, crypt width, mucosal thickness, submucosal thickness, muscular layer thickness, and inflammatory infiltration, while colonic parameters included crypt depth, crypt width, mucosal thickness, muscular layer thickness, and inflammatory infiltration. All values are presented as mean ± SEM. a and b indicate $p < 0.05$ (a vs. SHAM group; b vs. S group).

	GROUPS				
	SHAM	S	SM	SF	SMF
**ILEUM**					
villus height	275.6 ± 4.8	262.1 ± 3.1[a]	254.4 ± 2.5	254.1 ± 1.3	252.8 ± 1.6[b]
villus width	64.2 ± 1.6	73.4 ± 3.5	68.7 ± 1.7	64.4 ± 1.6	69.2 ± 0.9
crypt depth	142.5 ± 2.6	145.2 ± 1.6	139.8 ± 1.1	146.5 ± 4.2	141.6 ± 1.8
crypt width	34.7 ± 1.0	39.3 ± 1.8	40.4 ± 2.1	40.1 ± 2.7	40.6 ± 2.4
mucosa	433.1 ± 15.0	419.4 ± 9.2	405.3 ± 6.2	420.0 ± 3.7	413.8 ± 4.6
submucosa	28.3 ± 1.4	31.9 ± 2.2	28.6 ± 1.2	27.0 ± 1.5	27.8 ± 1.6
muscular layer	76.6 ± 2.4	77.4 ± 6.7	71.3 ± 2.9	76.3 ± 2.7	78.0 ± 4.3
inflammation	2.0 ± 1.2	9.7 ± 0.2[a]	8.7 ± 0.8	9.7 ± 0.2	10.0 ± 0.0
**COLON**					
crypt depth	156.5 ± 1.0	162.3 ± 2.4	165.3 ± 3.7	155.5 ± 2.5	164.1 ± 4.0
crypt width	40.0 ± 2.2	40.0 ± 1.4	38.9 ± 0.9	38.4 ± 1.4	41.5 ± 1.9
mucosa	162.0 ± 0.9	167.2 2.3	169.8 ± 3.2	169.0 ± 1.7	169.5 ± 3.2
muscular layer	144.1 ± 12.1	163.7 ± 8.1	151.2 ± 6.8	139.4 ± 12.4	160.8 ± 10.1
inflammation	0.0 ± 0.0	4.7 ± 1.4[a]	6.3 ± 1.8	5.0 ± 1.5	4.7 ± 0.9

Supplementary Table S2. Hepatic antioxidant enzyme activities and oxidative stress markers 24 hours after CLP-induced sepsis. Parameters included superoxide dismutase 1 (SOD1), superoxide dismutase 2 (SOD2), catalase (CAT), glutathione peroxidase (GSH-Px), glutathione S-transferase (GST), glutathione reductase (GR), total glutathione (GSH), sulfhydryl (SH) groups, lipid peroxidation (LPO), and protein carbonyls (PCO). All values are presented as mean ± SEM. a and b indicate $p < 0.05$ (a vs. SHAM group; b vs. S group).

	**SHAM**	**S**	**SM**	**SF**	**SMF**
SOD1 (U/mg tissue)	3419,2 ± 580,2	2317,5 ± 206,4	1923,4 ± 163,5	3307,1 ± 164,0[b]	3260,0 ± 154,9[b]

SOD2 (U/mg tissue)	2027,3 ± 145,7	2212,6 ± 82,8	2597,8 ± 116,0[b]	2390,7 ± 83,4	2658,7 ± 59,7b
CAT (µmol H_2O_2/min/g w.m.)	1331,6 ± 73,7	964,0 ± 125,7[a]	778,4 ± 52,1	660,4 ± 75,3	682,1 ± 54,5
GSH-Px (nmol NADPH/min/g w.m.)	62571,3 ± 3493,0	54766,9 ± 1910,0[a]	50355,5 ± 3550,4	54761,2 ± 3844,8	53985,1 ± 2823,8
GST (nmol GSH/min/g w.m.)	160287,8 ± 19969,6	156635,4 ± 13370,7	141968,8 ± 9090,3	186818,6 ± 10821,8[b]	181649,8 ± 11045,4
GR (nmol NADPH/min/ g w.m.)	9832,8 ± 473,7	11270,1 ± 627,4	9191,7 ± 388,7	9526,3 ± 266,2	8777,1 ± 280,6[b]
GSH (nmol/mg w.m.)	5494,6 ± 629,4	5969,4 ± 737,8	5334,0 ± 982,5	5542,3 ± 487,6	4986,5 ± 589,4
SH groups (µmol/mg w.m.)	3284,7 ± 164,8	3420,2 ± 104,7	3612,8 ± 298,9	3742,5 ± 114,3	4027,6 ± 249,1
LPO (nmol MDA/mg w.m.)	20,6 ± 2,0	23,2 ± 1,9	19,2 ± 1,3	18,2 ± 0,2	18,6 ± 0,2
PCO (nmol/mg prot)	41,8 ± 5,8	48,7 ± 4,1[a]	45,9 ± 5,0	54,9 ± 5,8	52,8 ± 3,4

Supplementary Table S3. Detailed statistical analyses for all biochemical, molecular, and histopathological parameters included in the study. For SHAM vs S comparisons, results are presented as p-values, Cohen's d, and 95% confidence intervals (CI). For comparisons among septic subgroups (S, SM, SF, SMF), results are reported as p-values, η^2 effect sizes, and 95% confidence intervals (CI). Effect size estimates and confidence intervals are provided to enable interpretation of the magnitude and precision of observed differences beyond statistical significance alone.

	**SHAM vs S**			**S vs SM vs SF vs SMF**		
	p-value	Cohen's d	95% CI	p-value	η^2	95% CI
**ADRENAL GLANDS**						
Noradrenaline	0.03447	1.26	0.09 – 2.37	0.459	0.06	0.1 – 0.30
Adrenaline	0.03924	1.16	0.06 – 2.21	0.3507	0.07	0.01 – 0.31
**SERUM**						
Total proteins	0.1986	0.61	-0.32 – 1.52	0.422	0.06	0.01 – 0.30
Albumin	0.4452	0.36	-0.53 – 1.24	0.268	0.09	0.02 – 0.34
Bilirubin	0.03349	-0.98	-1.90 – -0.03	0.2948	0.10	0.02 – 0.34
ALP	0.9447	0.04	-0.93 – 0.99	0.01818	0.16	0.05 – 0.42
ALT	0.1094	-0.71	-1.56 – 0.16	0.237	0.10	0.02 – 0.34
AST	0.7014	-0.18	-1.05 – 0.68	0.206	0.10	0.02 – 0.35
GGT	0.4241	-0.39	-1.24 – 0.46	0.463	0.06	0.01 – 0.30
LDH	0.8446	-0.09	-1.01 – 0.84	0.592	0.04	0.01 – 0.27
**ILEAL HISTOPATHOLOGICAL PARAMETERS**						
villus height	0.04307	1.61	-0.07 – 3.2	0.0262	0.38	0.14 – 0.68
villus width	0.07847	-1.42	-2.76 – 0.01	0.07276	0.30	0.1 – 0.62
crypt depth	0.3686	-0.59	-1.9 – 0.78	0.1679	0.18	0.04 – 0.55
crypt width	0.09117	-1.34	-2.66 – 0.05	0.975	0.01	0.01 – 0.36

mucosa	0.4308	0.52	-0.84 – 1.83	0.321	0.16	0.03 – 0.53
submucosa	0.246	-0.86	-2.10 – 0.42	0.182	0.20	0.04 – 0.55
muscular layer	0.92	-0.07	-1.22 – 1.08	0.732	0.06	0.02 – 0.43
inflammation	0.01063	-4.58	-8.21 – 0.96	0.4491	0.2	0.05 – 0.56
**COLONIC HISTOPATHOLOGICAL PARAMETERS**						
crypt depth	0.1004	-1.31	-2.63 – 0.08	0.1792	0.23	0.06 – 0.56
crypt width	0.9902	0.01	-1.29 – 1.3	0.301	0.16	0.04 – 0.54
mucosa	0.1169	-1.24	-2.54 – 0.13	0.7589	0.05	0.01 – 0.41
muscular layer	0.1967	-0.89	-2.24 – 0.53	0.31	0.15	0.03 – 0.52
inflammation	0.02105	-1.92	-3.49 – -0.26	0.7905	0.04	0.01 – 0.41
**COLONIC EXPRESSION OF INFLAMMATORY MEDIATORS**						
TLR4	0.2559	-0.59	-1.59 – 0.42	0.854	0.03	0.01 – 0.32
TNFα	0.01476	-0.35	-1.33 – 0.64	0.624	0.10	0.02 – 0.42
IL1β	0.01107	-1.43	-2.69 – -0.10	0.9462	0.01	0.01 – 0.30
IL10	0.001	-1.43	-2.70 – -0.09	0.1834	0.16	0.04 – 0.48
Nrf2	0.4957	-0.35	-1.40 – 0.70	0.9003	0.04	0.01 – 0.34
Total NF-κB p65	0.01034	-0.40	-1.4 – 0.64	0.4158	0.05	0.01 – 0.31
HMGB1	0.6879	-0.18	-1.10 – 0.75	0.493	0.057	0.01 – 0.29
BAX/Bcl-2	0.8396	-0.12	-1.25 – 1.02	0.867	0.032	0.01 – 0.39
**HEPATIC EXPRESSION OF INFLAMMATORY MEDIATORS**						
TLR4	0.01796	1.43	0.24 – 2.57	0.68	0.08	0.02 – 0.43
TNFα	0.1087	-0.90	-1.95 – 0.19	0.1844	0.18	0.04 – 0.50
IL1β	0.01998	-1.06	-2.15 – 0.09	0.3510	0.40	0.18 – 0.66
IL10	0.03304	-1.29	-2.62 – 0.12	0.07623	0.32	0.11 – 0.63
Nrf2	0.006811	1.74	0.42 – 3.02	0.06607	0.24	0.08 – 0.54
Total NF-κB p65	0.9018	0.10	-0.83 – 1.04	0.9954	0.01	0.01 – 0.21
HMGB1	0.2375	0.72	-0.27 – 1.69	0.9182	0.04	0.01 – 0.27
BAX/Bcl-2	0.2147	-0.59	-1.53 – 0.38	0.1638	0.08	0.01 – 0.32
**HEPATIC OXIDATIVE STRESS MARKERS**						
SOD1 (protein)	0.2632	0.55	-0.50 – 1.57	0.1577	0.18	0.05 – 0.43
SOD1 (enzyme)	0.1313	0.73	-0.31 – 1.75	0.000001	0.52	0.35 – 0.70
SOD2 (protein)	0.9279	-0.04	-0.98 – 0.89	0.5384	0.08	0.02 – 0.34
SOD2 (enzyme)	0.2546	-0.53	-1.48 – 0.44	0.00141	0.32	0.14 – 0.55
CAT (protein)	0.0527	0.41	-0.59 – 1.39	0.2125	0.09	0.02 – 0.33
CAT (enzyme)	0.0003149	2.18	0.96 – 3.35	0.147	0.13	0.03 – 0.38
GSH-Px	0.05068	0.94	-0.09 – 1.94	0.781	0.03	0.01 – 0.25
GST	0.8762	0.07	-0.86 – 0.99	0.0399	0.19	0.05 – 0.45
GR	0.1062	-0.82	-1.73 – 0.11	0.01551	0.36	0.18 – 0.58
GSH	0.6477	-0.22	-1.11 – 0.67	0.754	0.03	0.01 – 0.27
SH groups	0.4764	-0.33	-1.26 – 0.62	0.1073	0.12	0.03 – 0.37
LPO	0.31	-0.42	-1.32 – 0.50	0.1012	0.25	0.09 – 0.50
PCO	0.04613	-0.46	-1.39 – 0.50	0.675	0.05	0.01 – 0.3
